# 

**Published:** 1875-07

**Authors:** 


CONTENTS.

| | PAGE. |
|---|---|
| Mental and Physical Suffering | 197 |
| Diseased Meats | 199 |
| Words to the Young | 200 |
| Measles and Scarlet Fever | 202 |
| Poisoning by Matches | 204 |
| Rheumatism and Dyspepsia | 204 |
| Our Summer at Maplewood. Chap. vi—Breakfast Table Gossip | 205 |
| The Teeth | 210 |
| Health and Religion | 211 |
| The Dry Earth System | 212 |
| How to be Acceptable | 214 |
| Self-Remedied | 215 |
| Tar in Skin Diseases | 218 |
| Medical Electricity | 219 |
| The Art Preservative | 220 |
| Insanity in Horses | 221 |
| Where shall We Spend the Summer | 222 |
| Heart Disease: Its Causes and Treatment | 225 |
| Granulated Wheat Biscuit | 227 |
| Another Tooth-Ache Remedy | 228 |
| Electricity and Life | 228 |
| A Wrong Righted | 231 |
| Household Gods | 234 |
| "Our Home" Dansville | 235 |
| Fuchsin as an Antiseptic | 235 |
| Side Board Refrigerator | 236 |
| New Cure for Piles | 237 |
| Care of the Eyes | 237 |

